# A mathematical reason for FEV1/FVC dependence on age

**DOI:** 10.1186/1465-9921-13-57

**Published:** 2012-07-04

**Authors:** Tomasz Gólczewski, Wojciech Lubiński, Andrzej Chciałowski

**Affiliations:** 1 Nałęcz Institute of Biocybernetics and Biomedical Engineering, Polish Academy of Sciences, Ks. Trojdena st. 4, 02-109, Warsaw, Poland; 2 Military Institute of Medicine, Warsaw, Poland

**Keywords:** Age-dependence, Forced expiratory maneuver, Lung function tests, Obstructive lung disease, Spirometry

## Abstract

**Background:**

Recent studies have showed that FEV1/FVC describing correspondence between the forced expiratory volume in one second (FEV1) and forced vital capacity (FVC) depends significantly on age. However, the nature of this dependence is uncertain. The study aim is to analyze mathematically the relationship between FEV1 and FVC to find a cause of the FEV1/FVC dependence on age in healthy subjects.

**Methods:**

The relationship was examined for 1,120 males and 1,625 females – Polish (Caucasian) population, healthy, never-smoking, aged 18 – 85 years, who performed a technically adequate spirometry maneuver. Lung functions were measured using the LungTest1000 (MES, Poland) with maximal effort according to the ATS/ERS guidelines.

**Results:**

A very strong, age-independent linear relationship between FEV1 and FVC was found in healthy individuals (the correlation coefficient r = 0.96). It can be described with the equation FEV1 = A x FVC + C, where A = 0.84 and C = −0.23 (−0.36) for females (males). As C is different from zero, FEV1/FVC depends on FVC because FEV1/FVC = A + C/FVC, in average. And thus, since FVC is significantly age-dependent, FEV1/FVC has to be also age-dependent because of the term C/FVC. In particular, the smaller the FVC value because of advanced age, the more significant the fall of FEV1/FVC.

**Conclusions:**

FEV1/FVC dependence on age in healthy individuals is of mathematical rather than biological nature. Due to the strong correlation between FEV1 and FVC in healthy subjects, the difference between patient’s FEV1 and the FEV1 value expected for patient’s FVC seems to be a more natural, age-independent description of the correspondence between patient’s FEV1 and FVC.

## Background

Spirometry is the fundamental diagnostic method in obstructive lung diseases because it is easy and inexpensive to perform, and thus it can be used as a screening test. If the expiration is forced, the values of spirometric indices are almost independent of patient’s activity; they depend only on the properties of the respiratory system because of the airflow limitation phenomenon [1-3]. Evaluation of forced spirometry results begins with analysis whether bronchial airflow capacity quantified by means of the forced expiratory volume in one second (FEV1) corresponds to the lungs size estimated with the forced vital capacity (FVC) or is too small. According to the present recommendations, the relationship between FEV1 and FVC is quantified by means of the ratio of these indices, i.e. FEV1/FVC, introduced by Tiffaneau and Pinnelli [4] about 60 years ago. If the value of FEV1/FVC is smaller than its lower limit of normal (LLN), a bronchial obstruction should be diagnosed.

In previous GOLD recommendations [5], irrespectively of the age of examined subjects, the constant value of FEV1/FVC equal to 70% was assumed as the LLN to diagnose the obstruction in chronic obstructive pulmonary disease, despite that existence of some dependence of FEV1/FVC on age had been known. However, more recent studies have shown that FEV1/FVC decreases with age so significantly [6-8] that age-dependent LLN of FEV1/FVC has to be taken into account [9].

Many authors have analyzed how FEV1/FVC changes with age and which regression equation is the best among others [6-8,10-14]. Accurate matching of the equation is essential since an incorrect equation may lead to either the abandonment of a sick individual or the diagnosis of a non-existing disease and unnecessary treatment of healthy individuals. Unfortunately, the present equations proposed by various authors differ significantly in predictions, especially for more advanced age, i.e. for such age range for which incidence of obstructive lung diseases rises. Moreover, the constant value equal to 70% versus age-dependent LLN is still discussed [15].

Because of such meaning of FEV1/FVC, the problem “why it depends on age” should be also studied. This paper contains a mathematical study because the value of FEV1/FVC is not any directly measured quantity; it is a mathematical index calculated from two quantities that are measured. Therefore, the following doubt might appear: is it possible that the age-dependence of this index is an accidental effect of its calculation method not matched to the nature of the true relationship between FEV1 and FVC? To test this hypothesis, a direct statistical analysis of the relationship in healthy individuals was performed.

The nature - whether mathematical or physiological - of troublesome dependence of an index that is calculated (not measured) on some factors may be very important: if the nature is mathematical then another, better mathematical index could be proposed, which is the additional aim of this work.

## Material and methods

### Material

5130 females and 4716 males were examined within the project “Hope for Lungs” which was conducted in 2002–2005 by the Military Institute of Medicine, Warsaw, Poland. With the permission of the Local Ethics Committee, the authors utilized the database of that project in the analysis of the relationship between FEV1 and FVC that is presented in this paper. It should be noted that the same database was utilized in our other works related to different matters: prediction equations of a novel form [7] and an index for quantitative assessment of correctness of the flow-volume curve [16]. That project involved spirometry screening for obstructive lung diseases in Poland. With a mobile laboratory, the examinations were performed at 93 sites: both large cities and small towns as well as villages throughout Poland. For all subjects, after interview and written consent from participants, the examination was performed in sitting position using the same, regularly calibrated spirometer (LungTEST1000 by MES, Poland), in summer (between May and September), from 9 am to 4 pm. All examinations were performed and analyzed by the same group of six qualified employees performing routinely spirometry in the Central Clinical Hospital of the Polish Ministry of National Defense.

Like during elaboration of prediction equations, only data for healthy subjects were taken into account. Therefore, the following subjects were excluded from the analysis (exclusion criteria): persons younger than 18 year and older than 85 year, smokers, those reporting the occurrence of a chronic cough or dyspnea within the last 12 months, as well as individuals who were unable to perform spirometry properly (ATS [17] recommendations were used in the technical evaluation). The final selection was performed by the authors taking into account both medical and technical criteria. 3505 females and 3596 males were rejected due to failure to meet these criteria. Consequently, the results for 1625 females and 1120 males were used in the analysis. Table 1 presents details concerning these groups.

**Table 1:** Reference sample selection criteria and characteristics of the study sample

	**Males**	**Females**
Participants:	(n)	(n)
Total	4716	5130
Less age outside the range	4293	4767
Less ever smoking	2385	3382
Less invalid or irreproducible data	1454	1825
Less non-healthy subjects	1120	1625
Age in years	(n)	(n)
18–45	311	316
46–65	472	766
65–85	337	543
Age (yrs)	55 ± 16	57 ± 14
Height (cm)	173 ± 7	161 ± 6
FEV1 (L)	3.4 ± 1.0	2.4 ± 0.7
FVC (L)	4.5 ± 1.2	3.1 ± 0.8

Data are presented as the number of participants (n) or mean ± SD. FEV1- forced expiratory volume in one second; FVC- forced vital capacity.

## Data analysis

The strength of the relationship between FEV1 and FVC was described quantitatively with the fraction of explained variance (R^2^) and the correlation coefficient (r). As the correlation coefficient appeared very high, the relationship could be described accurately with a linear equation. Therefore, the linear regression of FEV1 on FVC was done. Age was used as an additional independent variable of the regression to examine a possible increase in R^2^ suggesting age influence on the relationship between FEV1 and FVC. Calculations were performed with the computer system *Statistica* (StatSoft, USA).

## Results

**Figure 1:**
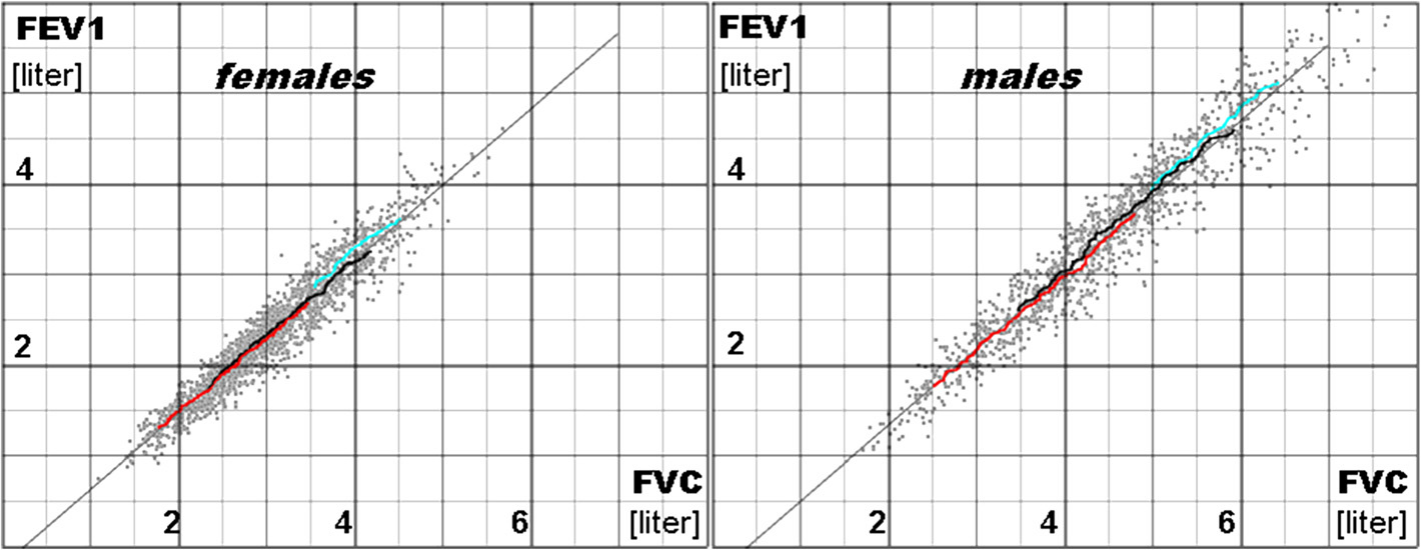
**Forced expiratory volume in one second (FEV1) versus forced vital capacity (FVC) in females and males.** Light gray dots – the studied material; thin lines – the linear regression; bold points creating curves – the moving 50th percentile of FEV1 for particular values of FVC calculated separately in three ranges of age (blue- 18–40, black- 41–65, and red- 66–85 years).

A very high correlation was evidenced between FEV1 and FVC, demonstrating thus a strong linear relationship (r = 0.96 both for males and females) (Figure 1). The assumed equation form for the linear regression of FEV1 on FVC was as follows:

(1) FEV1=A×FVC+C

The values of the coefficients **A** and **C** for males and females are shown in Table 2. The R^2^ coefficient is equal to 93% for males and 92% for females, which means that only 7–8% of the FEV1 variance cannot be explained with the FVC variability. R^2^ increased very little when age was added as the second independent variable in the regression of FEV1 (Table 2), which means that the relation between FEV1 and FVC is practically age-independent. Although age and height significantly influence both FEV1 and FVC, they have a very insignificant influence on the relationship between them (Figure 1). For example, if an average healthy woman has FVC = 3.5 L, she has FEV1 approximately equal to 2.75 L, whether she is young and short (the left end of the blue line in Figure 1) or of middle age and height (the middle of the black line in Figure 1) or is tall but older (the right end of the red line). Thus, the main result of the work could be expressed as follows: in healthy subjects FEV1 depends on FVC without respect to the other factors, including age and height.

**Table 2:** Linear regression of FEV1 on FVC and on FVC and age

	
FEV1 on FVC	FEV1 on FVC and age
females	females
FEV1 = 0.84·FVC – 0.23	FEV1 = 0.77·FVC + 0.28 – 0.0052·age
R^2^ = 92.0%, r = 0.96	R^2^ = 92.7%
males	males
FEV1 = 0.84·FVC – 0.36	FEV1 = 0.77·FVC + 0.32 – 0.0069·age
R^2^ = 93.1%, r = 0.96	R^2^ = 93.6 %

FEV1 (liter)- forced expiratory volume in one second; FVC (liter) - forced vital capacity; r- the correlation coefficient, R^2^ – fraction of explained variance. For all values shown in the Table p < 0.0001.

If FVC for an individual is known, the expected value of FEV1 can be estimated with the formula *1* using the coefficients from the Table 2. If this individual is healthy, such an expected value of FEV1 should be approximately equal to his/her real FEV1 since the correlation between FEV1 and FVC is so high. If, however, a patient is suffering from an obstructive lung disease, the difference between the measured and expected values of FEV1 should be significant.

Knowing patient’s FVC and making a modification of the formula *1*, also his/her FEV1/FVC might be approximated:

(2) FEV1/FVC=A+C/FVC

**Figure 2:**
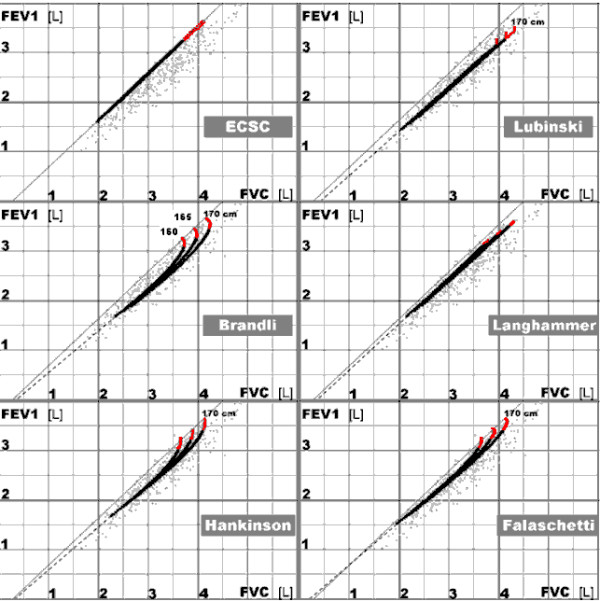
**A graphical interpretation of FEV1/FVC decline with age in healthy subjects.** FEV1 and FVC for an individual can be represented by one point on the plane determined by the coordinate system: FVC and FEV1. The ratio FEV1 to FVC is equal to the tangent of the angle α contained between the horizontal axis (FVC-axis) and the vector leading out to that point from the coordinate system origin. Thus, the lower value of the angle, the smaller the FEV1/FVC value.

The formula *2* consists of the constant term **A** and the term **C**/FVC, which is inversely proportional to FVC. Thus, since FVC depends significantly on age, also FEV1/FVC should depend on age, despite that the relation between FEV1 and FVC is age-independent. Figure 2 shows graphical interpretation of the above.

## Discussion

Since both FEV1 and FVC depend on a common factor that is age [7,8,11-14], one may expect some statistical correlation between FEV1 and FVC. Moreover, assuming that dependence of FEV1 and FVC on age in middle-aged and elderly healthy subjects is linear [7,11], also the relationship between FEV1 and FVC should be linear. Direct analysis of this relationship confirmed the linearity (formula 1), however the correlation between FEV1 and FVC appeared to be surprisingly high. In particular, regression of FEV1 on FVC produced R^2^ being much higher than R^2^ obtained during regression of FEV1 or FVC on age for the same sample of the Polish population [7].

The relationship between FEV1 and FVC can be described precisely by means of a linear equation with the constant term **C** different from zero. Such a value of the term C is a result of small differences in the relative rates of FEV1 and FVC decline with age. It can be proved that if the dependence of FEV1 and FVC on age is linear within some age range then:

(3) $$\text{C}=-\left({\text{S}}_{\text{FEV}1}-{\text{S}}_{\text{FVC}}\right)\times {\text{FEV}1}_{0}/{\text{S}}_{\text{FVC}}$$

where: FEV1_0_ is the mean value of FEV1 for the left end of this age range, S_FEV1_ and S_FVC_ are the decline rates of FEV1 and FVC, respectively (S_FEV1_ = 1.070%/year and S_FVC_ =0.992%/year in Polish females, S_FEV1_ = 1.098%/year and S_FVC_ =0.968%/year in males [7]).

**Figure 3:**
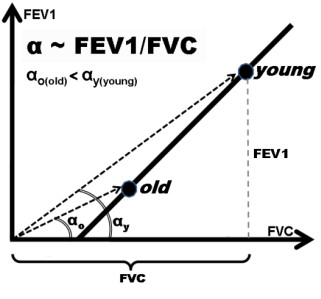
**Predicted forced expiratory volume in one second (FEV1) versus forced vital capacity (FVC) for females.** Light gray dots – the studied material. Bold curves – the mean values of FEV1 and FVC predicted for females aged 20 to 80 year (height = 160, 165, and 170 cm) with equations of various authors: ECSC [11], Lubinski and Golczewski [7], Brandli et al. [12], Langhammer et al. [13], Hankinson et al. [14], Falaschetti et al. [8]. The red parts of the curves correspond to data for females younger than 35 years (they are partly covered by the black parts).

Neither original data used by other authors [8,11-14] could be utilized to verify the existence of the constant term being different from zero nor their equations could be used in the formula 3 because those equations had forms being physiologically uninterpretable. However such a value of **C** appeared when values of FEV1 and FVC predicted by those equations were used (Figure 3). Note that all the curves in Figure 3 suggest the x-intercept different from zero, despite that some of these curves are not appropriate for the population analyzed here. Comparison of the relationship between values predicted with old ECSC equations [11] (thin continuous lines in Figure 3) with the relationships between FEV1 and FVC for subjects in this study as well as predicted with more recent equations (the other lines in Figure 3) suggests movement of the relationship to the right in comparison with ECSC (a possible reason: less restrictive criterion for the forced expiration time in the past causing underestimation of FVC [7,8,18]).

If the constant term **C** was equal to zero, FEV1 would be approximately proportional to FVC, i.e. FEV1 = A×FVC. Then, neglecting inter-individual differences, the FEV1/FVC index being the quotient of FEV1 and FVC would have the constant value equal to the proportionality constant **A**, approximately equal to the FEV1/FVC predicted value for the average young subject. However, because the **C** value is different from zero, an additional term, which is equal to **C**/FVC, appears in the expression defining the average value of FEV1/FVC (formula *2*). Since **C** is negative (Table 2), this term is subtracted from **A**, which makes FEV1/FVC lower than it would be without this additional term. It should be noted that Burrows et al. [19] observed some FEV1/FVC dependence on FVC in 1983.

As age influences significantly FVC, it also influences FEV1/FVC because of the **C**/FVC term, and thus age-dependence of FEV1/FVC is mainly an effect of the method of calculation, not a symptom of some direct, physiological influence of age on the relationship between FEV1 and FVC. Certainly, a physiological influence probably also exists but it is relatively small (Figure 1, Table 2). Among others, it can be related to differences in the onset age of FEV1 and FVC declines with age [7]; hence a nonlinearity in the relationship for youngest subjects in Figure 3.

Summarizing, FEV1/FVC, being a quantitative description of the relationship between FEV1 and FVC, depends on age in the middle-aged and elderly healthy subjects despite that this relationship *per se* is independent of age in those subjects. It means that FEV1/FVC is not a good index for quantifying the relationship because it suggests not existing dependence.

According to the current recommendations, FEV1/FVC is of key significance in the initial diagnosis of obstructive diseases. Its value decides whether the examined individual should be treated as suffering from obstructive lung disease or not. Therefore, the value of its LLN predicted for a patient has to be determined accurately. Comparison of regression equations proposed by different authors (e.g. [6-8,10-14]) shows that the mean values and LLN of FEV1/FVC in the Caucasian elderly significantly depend on the author; e.g. the mean value varies between 70% and 80%. Hence it appears that FEV1/FVC improperly describes the relationship between FEV1 and FVC as well as its really accurate values are difficult to determine.

As has been shown, FEV1 is strongly connected with FVC without respect to such factors important in spirometry as age and height. Therefore, from the clinical point of view, it would be convenient to simply assume that in healthy persons FVC determines FEV1 without respect to various factors. The authors realize that FEV1/FVC is a very established spirometric index. Since, however, it is a neither mathematically correct nor very reliable index, the authors would like to propose a new index to assess whether patient’s FEV1 corresponds to his/her FVC like in healthy subjects or it is too small. As the relationship between FEV1 and FVC is so strong and almost independent of age (esp. in the middle-aged and elderly), the authors suggest to use this relationship directly. According to our idea, the new index (called initially SAI - spirometric aberration index) would be calculated with the following formula:

(4) SAI=FEV1m−FEV1p

where FEV1m is the measured patient’s FEV1 whereas FEV1p is the FEV1 value expected for the measured patient’s FVC, i.e. FEV1p is the FEV1 value predicted using the Equation (1) with coefficients shown in Table 2 (for Polish population; other ethnic groups should have own coefficients). Thus:

(5) SAI=FEV1m−A×FVC−C

Note that SAI - like FEV1/FVC – can show whether patient’s FEV1 is too small in relation to the one predicted by his/her FVC. Additionally, it might suggest the obstruction severity.

The mean value of SAI for all healthy subjects is equal to zero from the definition of the linear regression. Dispersion of SAI in healthy subjects depends weakly on the FVC value, and thus on age, since FVC depends on age. Dispersion fall with age seems to be comprehensible because relative rather than absolute changes of FEV1 have physiological meaning. For example, FEV1 decrease by 1 l from 2 l to 1 l is much more significant for patient’s state in comparison with the same absolute decrease but from 4 to 3 l.

As SAI dispersion depends weakly on FVC (or age), its LLN (SAI_LLN_) has to depend on FVC (or age), too. To make diagnosing simpler and easier, the authors propose to use the standardized SAI, i.e.:

(6) $$\text{SAIs}=-\text{SAI}/{\text{SAI}}_{\text{LLN}}$$

Such standardization is analogous to standardizing in mathematical statistics (to random variable normalization), however with LLN instead of the standard deviation. Thus, the mean value of SAIs is equal to zero, like in the case of SAI, while LLN of SAIs is equal to −1.

Note that LLN of SAI can be related to either age or FVC, i.e. LLN can be determined either for subjects of a particular age (like the present LLN of FEV1/FVC, FEV1, and the other spirometric indices) or for subjects with a particular value of FVC. Future clinical studies should show which LLN is better from the clinical point of view.

**Figure 4:**
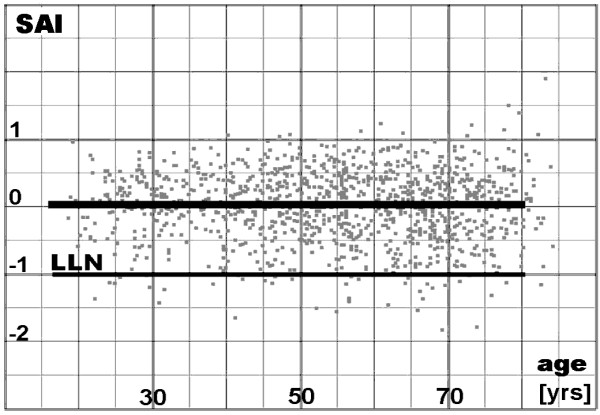
**Standardized Spirometric Aberration Index (SAI) plotted against age.** Gray dots - the studied material, the bold line - the predicted value (equal to SAI = 0), the thin line - the lower limit of normal (equal to SAI= −1).

Figure 4 presents dependence of SAIs on age for the analyzed population, when SAI_LLN_ (related to age) used in standardization (formula 6) were as follows:

(7) $${\text{SAI}}_{\text{LLN}}=0.0056\cdot \text{age}-0.800\;\text{for males}$$

(8) $${\text{SAI}}_{\text{LLN}}=0.0038\cdot \text{age}-0.553\;\text{for females}\text{.}$$

It should be stressed, that if severe obstruction decreases FVC, the SAI value may suggest a lower severity. However, the same problem concerns FEV1/FVC. Thus SAI is not worse than FEV1/FVC. Further analysis of data for patients suffering from obstructive lung diseases of various severity should show whether SAI is better than FEV1/FVC.

## Conclusions

Although age-dependence of both FEV1 and FVC is related to physiological ageing, FEV1/FVC dependence on age in healthy individuals is of mathematical rather than biological nature, i.e. in healthy persons FVC determines FEV1 without respect to other factors (including age) and FEV1/FVC is a mathematically wrong description of this relation between FEV1 and FVC. The difference between patient’s FEV1 and FEV1 predicted for patient’s FVC may be a more accurate and natural measure of correspondence between FEV1 and FVC.

## Competing interests

The authors declare that they have no competing interests.

## Authors’ contributions

TG conceived of the study, performed mathematical analyses, helped in technical evaluation of data, proposed a new index and drafted the manuscript; WL conceived the study, analyzed medical data and the new index, helped to draft the manuscript; AC continued WL’s work and prepared the manuscript. All authors read and approved the final manuscript.
